# Role of the Vanins–Myeloperoxidase Axis in Colorectal Carcinogenesis

**DOI:** 10.3390/ijms18050918

**Published:** 2017-04-27

**Authors:** Francesco Mariani, Luca Roncucci

**Affiliations:** Department of Diagnostic and Clinical Medicine, and Public Health, University of Modena and Reggio Emilia, Via Del Pozzo 71, I-41125 Modena, Italy; francesco.mariani@unimore.it

**Keywords:** vanins, myeloperoxidase, colorectal carcinogenesis, inflammation

## Abstract

The presence of chronic inflammation in the colonic mucosa leads to an increased risk of cancer. Among proteins involved in the regulation of mucosal inflammation and that may contribute both to structural damage of the intestinal mucosa and to intestinal carcinogenesis, there are myeloperoxidase (MPO) and vanins. The infiltration of colonic mucosa by neutrophils may promote carcinogenesis through MPO, a key enzyme contained in the lysosomes of neutrophils that regulates local inflammation and the generation of reactive oxygen species (ROS) and mutagenic species. The human vanin gene family consists of three genes: *vanin-1*, *vanin-2* and *vanin-3*. All vanin molecules are pantetheinases, that hydrolyze pantetheine into pantothenic acid (vitamin B5), and cysteamine, a sulfhydryl compound. Vanin-1 loss confers an increased resistance to stress and acute intestinal inflammation, while vanin-2 regulates adhesion and transmigration of activated neutrophils. The metabolic product of these enzymes has a prominent role in the inflammation processes by affecting glutathione levels, inducing ulcers through a reduction in mucosal blood flow and oxygenation, decreasing local defense mechanisms, and in carcinogenesis by damaging DNA and regulating pathways involved in cell apoptosis, metabolism and growth, as Nrf2 and HIF-1α.

## 1. Introduction

Vanins and myeloperoxidase (MPO) have a role in inflammation, metabolism and cellular stress, interplaying in various diseases, such as obesity, diabetes mellitus, and cancer, by regulating the migratory function of neutrophils, the first cells involved in the inflammatory processes, and pathways affecting oxidative stress and inflammation [1]. Moreover, the vanins–MPO axis may produce mutagenic compounds from endogenous and dietary elements, sustain a cyclic alternation of damage to epithelial barriers and proliferation, and regulate energetic, inflammatory and oxidative pathways, thus leading to cellular mutation and growth [2,3]. In this view, the vanins–MPO axis may be a central node in the regulation of colorectal cancer risk by integrating effects on diet, inflammation, and modifications of signaling pathways.

### 1.1. Myeloperoxidase

Myeloperoxidase (MPO) is the most abundant enzyme packed in azurophilic granules of the neutrophils, and can be released in the phagosome upon phagocytosis where it catalyzes the synthesis of hypochlorous acid (HOCl), from hydrogen peroxide (H_2_O_2_) and chloride ions (Cl^−^). Alternatively, the primary granules release their content into the extracellular milieu, and MPO can also be found in the site of inflammation causing damages to the host tissues. Thus, activated inflammatory cells induce necrosis in the surrounding tissue through oxidative stress mediated by the release of large amounts of proteinases and reactive oxygen and nitrogen species (ROS and RONS), hydrogen peroxide (H_2_O_2_) and hypochlorous acid [4,5].

Neutrophils affect colorectal carcinogenesis through ROS-independent and ROS-dependent mechanisms. Among the former, it should be mentioned the action on insulin receptor substrate-1 (IRS-1), and platelet-derived growth factor receptor (PDGFR) signaling that drives tumor cell proliferation, promotion of angiogenesis through the production of cytokines and angiogenic factors, and the regulation of both the innate and the adaptive immune responses through interactions with the other immune cells [6]. In this review, we will analyze the second mechanism that involves the MPO-mediated formation of HOCl. MPO can be used as a marker of inflammation in colorectal mucosa [7]. It has been reported that neutrophil infiltration and neutrophil-derived ROS correlated with DNA damage, DNA point mutations, DNA replication errors and higher DNA mutation frequency, leading to genetic instability, a hallmark of cancer [8,9].

### 1.2. Vanins Genes and Proteins

Several vanin genes have been identified, including three human sequences, *vanin-1*, *vanin-2*, and *vanin-3* with a similar structure and a high degree of homology of seven exons, mapped in a region on chromosome 6q 23–24 [10].

Vanins are pantetheinases, and have a nitrilase-like domain at the carboxyl-terminus, that hydrolyzes a carboamide linkage in d-pantetheine, thus providing release of pantothenic acid (vitamin B5) and cysteamine (CysH, 2-aminoethanethiol), which is in equilibrium with its oxidized form cystamine (CysN) [11]. The vanin-1 protein also has a so-called base domain, with a suggested role in binding to other proteins and in signaling, that regulates the enzymatic activity of vanins through allosteric movements [12]. *Vanin-1* and *vanin-2* genes have a region containing a glycosyl-phosphatidylinositol (GPI)-anchored cleavage site in exon 7 responsible for the attachment to the cell membrane through GPI anchoring [13,14]. These proteins may be released from cell surfaces as soluble forms through a cleavage by phospholipase C. In neutrophils, vanin-3 has nine splice variants lacking the full span of exon 7 [15].

Thus, vanin-1 is an ectoenzyme (GPI)-linked anchored to the cell surface, and is expressed by the spleen, thymus, lymph nodes, urethra, kidney, parts of the respiratory tract, liver, intestinal tract and myeloid cells as CD15+ granulocytes and CD14+ monocytes. The vanin-1 gene is preferentially expressed by epithelial cells [16,17,18].

The vanin-2 protein was originally called GPI-80 and can be found in both soluble and GPI-anchored, membrane-bounded forms. GPI-80/vanin-2 has pantetheinase enzymatic activity, but the activity is weaker than that of vanin-1. Vanin-2 (GPI-80/VNN2) is expressed by almost all tissues as colon, spleen, placenta, lung, and leukocytes, particularly neutrophils, where its expression increases during differentiation and maturation and has effect on mobility [19,20].

*Vanin-3*, by lacking the GPI-anchoring consensus, seems to encode a truncated protein and to be a secreted protein, and its expression is induced by oxidative stress [15,21].

## 2. Vanins–MPO Interplay in Inflammation Processes

### 2.1. Mac-1 as First Connection between Vanins and MPO

**Figure 1:**
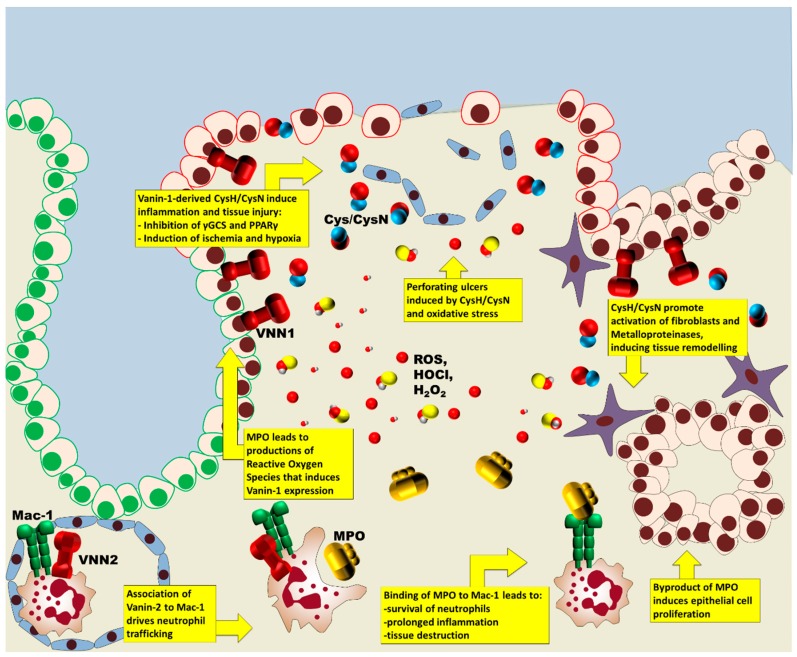
Vanins and myeloperoxidase (MPO) have both synergic and additive effects on inflammation of colonic mucosa. They regulate the processes of tissue destruction by driving and activating inflammatory cells into the inflamed sites, and inducing a prolonged inflammation and tissue lesions. Moreover, they also regulate subsequent remodeling, modulating the processes of angiogenesis, fibrosis and proliferation. ROS, reactive oxygen species; VNN1/2, vanin-1/2; γGCS, γ-glutamylcysteine synthetase; PPARγ, peroxisome proliferator-activated receptor γ.

Recently, it has been shown that MPO has pro-inflammatory properties also through the binding to macrophage-1 antigen (Mac-1) integrins, which are linked to neutrophil activation, thus acting independently from the enzymatic activity [22] (Figure 1). Mac-1 (CD11b/CD18, αMβ2 integrin, ITAM antigen) is a member of the β2 integrin family that mediates leukocyte adhesion and transmigration. It has been reported that Mac-1 has an oncogenic role during colorectal carcinogenesis, probably by promoting myeloid cell migration to the tumor sites in the colon that, through secretion of cytokines, may result in intestinal tumorigenesis [23,24].

However, extracellular MPO may bind to Mac-1 on the neutrophil membrane and modify intracellular signaling pathways, leading to phosphorylation of p38 MAPK, ERK 1/2 and PI3K, and to activation of NF-κB. This induces increased degranulation with release of elastase and MPO from the azurophilic granules, upregulation of surface expression of Mac-1 itself, and increased NADPH oxidase activity with superoxide production. The binding of MPO to Mac-1 also prevents mitochondrial dysfunction and activation of caspase-3, thus extending the life span of functional neutrophils, suppressing the cell death program and delaying the resolution of inflammation. An escape from neutrophil apoptosis is associated to non-resolving inflammation with tissue destruction. Thus, MPO through MPO–Mac-1 interaction can recruit, activate and sustain a prolonged survival of neutrophils independently of its catalytic activity, amplifying the inflammatory cascade, and activating proteolytic enzymes and oxidant products [25,26].

Vanin-2 has a role in inflammation, too, by regulating leukocyte adhesion and migration to inflammatory sites. Vanin-2 plays a role in neutrophil trafficking by physically associating in close proximity (≤7 nm) with Mac-1 on the human neutrophil surface, during the processes of adhesion and migration. Vanin-2 proteins are clustered on pseudopodia in the forward surface of activated neutrophils during attachment to the vessel wall, where they may increase the level of Mac-1 itself on the surface, thus facilitating the movement of migrating neutrophils to the wound site. Adherence of Mac-1 to ligands as fibrinogen and iC3b, or activation of β2-integrin by stimulants such as TNF-α and fMLP, leads to release of soluble vanin-2 [27,28,29].

### 2.2. Vanins and MPO as Key Players in Oxidative Stress Generation

Neutrophils may mediate wound healing but also sustain tumor proliferation, angiogenesis and metastasis. In the colonic mucosa, MPO activity correlates with the severity of colitis and is an indicator of colon cancer risk [7,30,31].

The free radicals into the cell cause lipid peroxidation and damages to DNA and to proteins, favoring carcinogenesis through the generation of DNA mutations, genomic instability, protein adducts and alterations in signaling pathways crucial to cellular functions, leading to malignant transformation [32,33,34].

The induction of various oxidative stress response genes is regulated by antioxidant response elements (AREs), found in the 5′-flanking region of the gene. Vanin proteins are tissue sensors for oxidative stress, reflecting inflammation severity linked to neutrophil activation as intestinal inflammation and experimental colitis. Vanin-1 expression is induced by exposure to oxidative stress and to cellular stressors as H_2_O_2_ and ROS. It has been reported the presence of two functional antioxidant response elements within the vanin-1 promoter, probably required for induction by H_2_O_2_, and a peroxisome proliferator-activated receptor (PPAR) response element in the promoter region of the gene, resulting in an amplification of inflammation and in fibrosis. Vanin-1 decreases the stores of reduced glutathione, promoting the inflammatory reaction and intestinal injury, mainly through cysteamine/cystamine (CysH/CysN, here referred to as Cys) [35,36,37,38] (Figure 1).

Cysteamine (mercaptoethylamine, HS-CH_2_-CH_2_-NH_2_) is a reducing aminothiol which induces duodenal perforating ulcers within 24–48 h when administered in high doses, by increasing gastric acid secretion [39]. However, it has also been observed a direct, necrotizing, cytotoxic effect for cysteamine, not related to gastric acid secretion, and not only in the stomach and duodenum, but also in other parts of the gastrointestinal tract, as the colon through several mechanisms including a reduction in mucosal blood flow, a decrease of local defense mechanisms, and an alteration in the redox state in the early pre-ulcerogenic mucosa. Cysteamine causes a decrease blood flow in the duodenum within 5–15 min after administration, probably by the local release of endothelin-1 (ET-1), a potent vasoconstrictor that causes tissue ischemia and hypoxia [40,41].

## 3. Role of Chemical by Products

### 3.1. Vanin-Derived Cysteamine

Cysteamine increases the expression and the activity of hypoxia-inducible factor 1α (HIF-1α) in the early pre-ulcerogenic phase after cysteamine administration, and this reaction claims tissue ulceration instead of wound healing. Cysteamine also causes a rapid induction of early growth response factor-1 (Egr-1), a hypoxia-associated protein, with the subsequent increase of growth factor production, including VEGF. Several studies support the hypothesis that Cys can regulate the redox status and reduce the oxygenation of the mucosa at an early stage of ulcer development. A marked neutrophil accumulation in the stomach and duodenum of cysteamine treated rats has also been reported [39,40,41,42].

**Figure 2:**
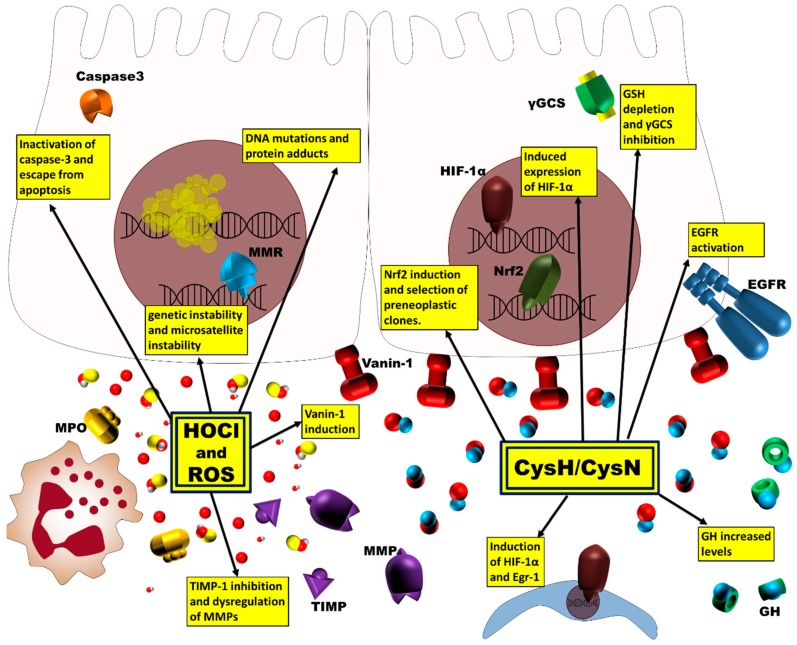
MPO and vanin-1, through their byproducts, modulate several pathways involved in carcinogenesis. They can sustain the generation of mutated clones, favor escape from apoptosis and selection of neoplastic clones, and drive overgrowth of cancer cells. MMP, metalloproteinases; GSH, glutathione; TIMP, tissue inhibitors of metalloproteinase; HIF-1α, hypoxia-inducible factor 1α; Egr-1, early growth response factor-1; GH, growth hormone; EGFR, epidermal growth factor receptor; MMR, mismatch repair; Nrf2, nuclear factor (erythroid-derived 2)-like 2.

Cys may inactivate many proteins and several enzymes as tissue transglutaminase and Caspase 3, targeting sulfhydryl groups of active site and disulfide bridge. Cys inhibits reduced glutathione (GSH) synthesis by inhibiting γ-glutamylcysteine synthetase (γGCS), the rate-limiting enzyme in the GSH synthesis, but also superoxide dismutase (SOD) and glutathione peroxidase (GSH-Px). Indeed, during the inflammation of colonic mucosa, it has been observed a mucosal GSH deficiency, caused by a decreased activity of γ-glutamylcysteine synthetase and γ-glutamyltransferase, two key enzymes in GSH synthesis [35,43,44,45] (Figure 2).

However, Cys administration leads to a robust initiation of nuclear factor (erythroid-derived 2)-like 2 (Nrf2)-driven transcription. Nrf2 is one of the key regulators of cellular defense against inflammatory damage and of antioxidant defense. Recently, it has been proposed an activation of Nrf2 as a mechanism of selection for preneoplastic clones of colon cancer [46,47,48].

In mouse models ovariectomy and old age can increase sensitivity to Cys-induced ulcerations, while estrogen administration is related to a decreased sensitivity to ulcer induction, suggesting that estrogens can protect from the development of cysteamine-induced duodenal ulcers [49]. These data provide interesting recalls to the protective role of estrogens in colorectal carcinogenesis.

Moreover, Cys increases gastric and plasma ghrelin levels, and causes a depletion of somatostatin. Ghrelin is a peptide with important physiological roles including the stimulation of growth hormone (GH) release, gastric motility, and the increase of food intake and body weight. Plasma ghrelin levels are significantly increased after Cys treatment, as well as in the pre-ulcerogenic phase, when no mucosal neutrophil accumulation or ulcer formation was observed. Somatostatin is a neuropeptide present in the gastrointestinal tract that can modulate lymphocyte function. Cysteamine is able to deplete somatostatin in the intestine. Thus, it has been hypothesized that Cys, by depleting somatostatin, may enhance inflammation in the mucosa. However, the depletion of somatostatin might be the main factor sustaining the development of cysteamine-induced ulcers, and treatment with somatostatin prevents Cys-induced ulcers formation and has an inhibitory effect on ghrelin secretion. The reduction in somatostatin levels caused by Cys administration is followed by an increased concentration of growth hormone, with stimulating effect on cells [42,50,51].

### 3.2. Involvement of Taurine and Taurine Chloramine

The free thiol cysteamine is a potentially intermediate in taurine biosynthesis, as it may be subsequently oxidized to hypotaurine by cysteamine (2-aminoethanethiol) dioxygenase (ADO), and further oxidized to taurine by hypotaurine dehydrogenase [52,53]. Taurine (2-aminoethanesulphonic acid), one of the most abundant sulfur-containing free amino acid in the body, is present in high concentrations in various mammalian cells, where it is essential for osmoregulation, membrane stabilization, neurotransmission, and cellular oxidative status by increasing GSH levels as well as cellular Ca^2+^ homeostasis [54]. Furthermore, taurine is one of the metabolites found to be more prevalent in colorectal cancer, using tissue metabolomics. It has been reported that taurine, cysteamine and cystamine are also present at higher levels in the serum of colorectal cancer patients as compared to healthy subjects, and that their levels are higher in patients with colorectal cancer at stages I and II with respect to those at stages III and IV [55,56].

Neutrophils, when recruited into the site of inflammation, generate a variety of highly reactive oxidants as hypochlorous acid (HOCl). An excess of hypochlorous acid may lead to tissue damage and development or progression of the disease. Taurine is released in large amounts by stimulated neutrophils, reacting with HOCl, and generating taurine chloramine (Tau-Cl; SO_3_(CH_2_)_2_NHCl). Thus, where MPO activity is increased, as in inflamed colorectal tissue and in preneoplastic lesions, there is a high generation of HOCl that converts taurine into taurine chloramine [57].

However, taurine also has anti-apoptotic properties, and Tau-Cl retains oxidative properties causing direct mitochondrial damage. This can refer to studies that reported both an increase of MPO and a decreased apoptosis since the early preneoplastic phases of colorectal carcinogenesis: in correspondence of an increase of MPO-positive cells, large amounts of taurine may be released into the environment, reducing apoptosis. It has also been hypothesized that Tau-Cl activates EGF receptor driven by oxidation of a cell surface target. Tau-Cl, HOCl and other reactive oxygen species have specific and different cellular effects and action on MAPK activation [7,58,59].

## 4. Interplay in Colorectal Cancer Pathways

Intestinal epithelial cells are the first line in the innate defense of the intestine, and an alteration in their functions or architecture is a feature of colitis, as in inflammatory bowel diseases (IBD), and cancer. Furthermore, epithelial cells are able to produce pro-inflammatory signals, playing a key role in the early events of tissue inflammation. In the gut, vanin-1 is highly expressed by enterocytes where promotes tissue injury by inducing oxidative stress, mucosal ulceration, inhibiting the expression of peroxisome proliferator-activated receptor γ (PPARγ), a negative regulator of NF-κB with anti-inflammatory activity in the intestine, and by increasing the expression and the release of pro-inflammatory cytokines and epithelial molecules, thus controlling gut immune responses and the development of acute colitis. A persistent inflammation and ulceration as caused by Cys, is a risk factor associated with an increased susceptibility to develop colon cancer. As previously reported, vanin-1 decreases the stores of reduced glutathione acting as glutamine analogue, by interacting with a sulfhydryl group at a second site of the target enzyme and mimiking the negative feedback regulation exherted by GSH. GSH depletion is related to inflammatory and tumoral pathways as p21ras, mitogen activated protein (MAP) kinase, and NF-κB [35,36,60,61]. Recently, it has been reported that vanin-1 production of Cys may be a central mechanism responsible for cell growth and tumorigenesis in the colon [62]. Cys also promotes activity of matrix metalloproteinases (MMPs), a family of zinc endopeptidases, involved in tissue remodeling and in many human diseases, including cancer and tissue ulceration. Moreover, Cys regulates HIF-1α activity increasing the interactions of HIF-1α with others transcription factors. HIF-1α is a factor with various roles in colon carcinogenesis, including adaptation to hypoxia, proliferation and angiogenesis [18,63,64] (Figure 2).

In colon cancer, interleukin-6 (IL-6) is a tumor-promoting factor by inducing neoplastic cell proliferation through activation of the STAT3 oncogene in dysplastic epithelial cells, and its levels correlates with tumor size. In colonic tumors, lack of vanin-1 is associated to higher levels of PPARg and to a reduction in IL-6 production and STAT3 activation. Thus, vanin-1 effects on proliferative potential of enterocytes may be exerted through IL-6. However, vanin-1 deficiency may also limit the development of colon cancer by down-regulating several mediators of inflammation in intestinal epithelial cells that promote colorectal carcinogenesis and are overexpressed in tumor as COX-2, iNOS and MMP9 [18].

Pantothenic acid is a profibrotic agent that may increase and accelerate the wound-healing processes by recruiting migrating fibroblasts to the affected areas and promoting the proliferation and activation of fibroblasts, and collagen synthesis [65,66]. However, a prolonged and not equilibrated succession of proliferation and death can lead to erosion of the epithelium, and thus to lack of function of the physical barrier.

Inflammation in the normal colonic mucosa, observed in colitis, causes overproduction of reactive oxygen species and subsequent tissue injuries. A great variety of modified DNA molecules are produced by the interaction with ROS, especially 8-oxo-7,8-dihydro-2,-deoxyguanosine (8-OH-dG), and lack of adequate 8-OH-dG repair may lead to carcinogenesis. Oxidative DNA damage accumulates in colorectal mucosa of patients with IBD, and mucosal 8-OH-dG concentrations increase with the duration of the inflammation and with dysplasia in these patients. The enzyme for repair of 8-OH-dG in the DNA molecule is the human homolog of the base excision repair gene MutY (MYH), a DNA glycosylase related to a hereditary form of colonic polyposis. Furthermore, oxidative DNA damage derived from exposition to hydrogen peroxide can induce microsatellite instability (MSI) [67,68,69].

Hydrogen peroxide also contributes to the activation of HIF-1α and NF-κB, a master regulator of the innate immune response, to damage DNA, cellular membranes and organelles, and to convert fibroblasts into activated myofibroblasts, favoring colon cancer development [70].

MPO may act not only as a bactericidal enzyme through the formation of hypochlorous acid, but may also regulate several cell signaling pathways. MPO, together with iNOS, can nitrosylate and inactivate caspase-3, thus allowing the escape from apoptosis for transformed cells. It has been reported that the MPO-mediated production of low level of hypochlorous acid may modulate the activity of mitogen-activated protein (MAP) kinases, transcription factors, tumor-suppressor proteins and metalloproteinases. At sites of inflammation, HOCl generated by MPO oxidizes Cys residues of TIMPs (Tissue inhibitors of metalloproteinases) abrogating TIMP-1 inhibitory activity during inflammation and dysregulating MMPs activation, thus affecting colorectal carcinogenesis [59,71,72]. It has been reported that ROS derivatives and hydrogen-peroxide (H_2_O_2_) induce PGC-1a (PPARg coactivator 1a), a key regulator of anti-oxidative defense program, that may have a central role in inducing colon carcinogenesis and promoting tumor growth [73,74].

## 5. Interaction of Vanins–MPO with the Environment and Metabolism

Epidemiological studies suggest that nutritional factors such as red and processed meat, animal fat and ethanol may be associated with a higher risk for the development of colorectal cancer, probably through the presence of genotoxic carcinogens in the gut lumen, derived directly by ingested foods or produced endogenously as metabolites. These risk factors include heterocyclic amines, polycyclic aromatic hydrocarbons and heme, which has toxic and genotoxic actions [75,76]. Among these compounds, ferric iron is a particular risk factor for colon cancer for its ability of forming reactive oxygen species via the Fenton reaction.

Thus, neutrophils act on colorectal carcinogenesis also influencing the complex effects of diet on colon cancer risk by the endogenous generation, through the MPO action, of acrolein, an oxidative by-product derived from unsaturated fats, serine, or threonine. Acrolein is one of the mutagenic and DNA-damaging oxidants generated by MPO, that forms protein adducts associated with a malignant progression in the colonic tissue. It has been reported that, in the colonic mucosa, acrolein augments colon tumor occurrence also by forming a protein adduct with PTEN (phosphatase tensin homolog), a prominent intestinal tumor suppressor, resulting in the activation of Akt kinase, a proto-oncogene that leads to cell growth and survival [77,78].

High iron exposure is related to increased cell proliferation in the intestinal crypts, promoting colorectal carcinogenesis. Some authors reported that perforating duodenal ulcers induced by oral administration of cysteamine may be exacerbated by elevated levels of endogenous iron. Conversely, iron-deficient diet decreased cysteamine-induced duodenal ulcers. Some studies reported that the cytotoxic effect of cysteamine depends on the generation of H_2_O_2_ in the presence of transition metals such as ferrous iron, Fe^2+^, and by subsequent generation of hydroxyl radicals. Thus, it has been proposed that the cytotoxic effect of cysteamine depends on the generation of thiols-derived H_2_O_2_ [60,79].

Iron exposure may have a genotoxic potential since colorectal preneoplastic lesions, contributing to cancer risk during the early stages of colorectal carcinogenesis. These data connect the vanin-derived Cys production with epidemiological studies showing that red meat consumption is associated with a compensative hyperproliferation of colonic epithelial cells, thus enhancing colorectal cancer risk, and vanin-1 may play a central role in this process [18,80,81].

## 6. Inhibition of the Vanins–MPO Axis

Lack of vanin-1 also decreases the levels of several genes associated with intestinal inflammation, as MIP-2, a local chemoattractant for neutrophils, and is thus associated with a concomitant reduced MPO activity [61].

In colonic tumors, lack of vanin-1 is associated to higher levels of PPAR and to a reduction in IL-6 production and STAT3 activation, whose levels correlate with tumor size [82]. However, vanin-1 deficiency may also reduce colon cancer development by down-regulating several mediators of inflammation in intestinal epithelial cells that promote colorectal carcinogenesis and that are overexpressed in tumors, as COX-2, iNOS and MMP9.

It has been reported that the lack of pantetheine hydrolase activity, as demonstrated in vanin-1 null mice, shows an enhanced γ-glutamyl-cysteinyl synthase (GCS) activity and thus elevated endogenous glutathione (GSH) levels in tissues. Thus, vanin-1 deficiency is associated with lower ROS concentrations and oxidative damage, and with a milder inflammation, increased resistance to oxidative stress and higher reconstitution rate due to reduced inflammation [11,21,61].

An inhibition of vanin-1 may be protective against intestinal inflammation and injury also through the prevention of the vanin-related release of proinflammatory cytokines from the intestinal epithelium. In this view, vanin-1 inactivation may favor the accumulation of cytoprotective pantethine and abrogate the profibrotic and oxidant effects of pantothenic acid/cysteamine [18,36,83,84].

### Targeted Compounds

The perspective of reducing colorectal cancer incidence through a chemoprevention approach is still missing. One of the main obstacles for the use of substances for chemoprevention is the lack of an effective target. The modulation of the vanins–MPO axis may be effective and useful for the prevention of cancer and of inflammatory diseases of the colon.

The inhibition of certain pathways regulated by the vanins–MPO axis in the treatment of colorectal carcinoma has been proposed. Somatostatin (SST) is a peptide with many effects on gastrointestinal function, i.e., suppressing gastrointestinal motility and regulating intestinal nutrient absorption and blood flow. SST can also directly decrease epithelial proliferation and induce apoptosis via its somatostatin receptor (SSTR). It has been proposed that a somatostatin analogue may be used in the therapy against advanced colorectal carcinomas [85]. Moreover, Mac-1 inhibition can be a potential target for colon cancer treatment, inhibiting angiogenesis and tumor growth [24].

MPO is associated with increased risk for various cancers, including lung adenocarcinoma [86]. Specific inhibitors of MPO may inhibit its activity in the tissues, preventing the damage. Among the compounds with anti-MPO activity, there are flavonoids, polyphenols, and melatonin [87,88,89].

Moreover, a new biologic tripeptide inhibitor of MPO activity, *N*-acetyl lysyltyrosylcysteine amide (KYC), has been proposed as a treatment during the inflammatory stage in the early stages of tumor development, suggesting the use of MPO inhibitors in cancer prevention [90,91].

PF-1355 (2-[6-(2,5-dimethoxyphenyl)-4-oxo-2-thioxo-3,4-dihydropyrimidin-1(2*H*)-yl]acetamide) is another novel selective MPO inhibitor that blocks HOCl formation. It has been proposed for the treatment of vasculitis where it is efficacious in reducing edema, neutrophil accumulation, production of proinflammatory cytokines and inflammation [92,93].

Another new, safe and well tolerated selective and irreversible inhibitor of MPO, named AZD3241, reduces the formation of excessive levels of reactive oxygen species contributing to reduce a sustained inflammation [94].

Aromatic hydroxamates as trifluoromethyl-substituted compound HX1, are described as reversible inhibitors of MPO with high potency and specificity, that physically block the active site inhibiting the halogenation activity of MPO. HX1 has been considered an efficient type of inhibitor, avoiding a permanent blockade of the enzyme and the generation of radical by-products [95].

Indeed, a MPO complete depletion may be an undesirable action, as reported by abrogation studies, since it has been associated with atherosclerosis and a slight increase of tumor formation [78,96,97]. Thus, a modulation of MPO activity may be the successful strategy for cancer prevention.

The pantetheinase activity of vanin-1 could be a target for the development of new anti-inflammatory compounds. Recently, several inhibitors of the vanin proteins have been developed. High-throughput approaches are used in order to identify pantetheinase inhibitors, but, until now, almost all of the compounds were nonselective and with modest potency, making them not useful. However, a new compound, named RR6, has shown a good bioavailability and pharmacodynamic profile as vanin inhibitor [98,99,100].

## 7. Conclusions

Vanins interact with MPO in modulating several pathways that affect the structure and function of the intestinal epithelium. They stand at the interface between inflammation and carcinogenesis, and represent interesting molecules to be investigated for possible preventive or therapeutic strategies in colorectal carcinogenesis. A concurrent modulation of these proteins may have the advantage of providing a synergistic action against carcinogenesis without, however, totally inhibiting pathways which are needed for physiological functions.

## Abbreviations

ROS	Reactive oxygen species
MPO	Myeloperoxidase
HOCl	Hypochlorous acid
CysH	Cysteamine
CysN	Cystamine
GPI	Glycosyl-phosphatidylinositol
Cys	Cysteamine/cystamine
HIF-1α	Hypoxia-inducible factor 1α
γGCS	γ–Glutamylcysteine synthetase
GSH	Glutathione
ADO	Cysteamine (2-aminoethanethiol) dioxygenase
TN-Cl	Taurine chloramine
PPARγ	Peroxisome proliferator-activated receptor γ
MMPs	Matrix metalloproteinases
8-OH-dG	8-Oxo-7,8-dihydro-2′-deoxyguanosine
TIMPs	Tissue inhibitors of metalloproteinases
VNN1	Vanin-1
